# The Effect of Chemical Amendments Used for Phosphorus Abatement on Greenhouse Gas and Ammonia Emissions from Dairy Cattle Slurry: Synergies and Pollution Swapping

**DOI:** 10.1371/journal.pone.0111965

**Published:** 2015-06-08

**Authors:** Raymond B. Brennan, Mark G. Healy, Owen Fenton, Gary J. Lanigan

**Affiliations:** 1 Civil Engineering, National University of Ireland, Galway, Co. Galway, Rep. of Ireland; 2 Teagasc, Environmental Research Centre, Johnstown Castle, Co Wexford, Rep. of Ireland; Catalan Institute for Water Research (ICRA), SPAIN

## Abstract

Land application of cattle slurry can result in incidental and chronic phosphorus (P) loss to waterbodies, leading to eutrophication. Chemical amendment of slurry has been proposed as a management practice, allowing slurry nutrients to remain available to plants whilst mitigating P losses in runoff. The effectiveness of amendments is well understood but their impacts on other loss pathways (so-called ‘pollution swapping’ potential) and therefore the feasibility of using such amendments has not been examined to date. The aim of this laboratory scale study was to determine how the chemical amendment of slurry affects losses of NH_3_, CH_4_, N_2_O, and CO_2_. Alum, FeCl_2_, Polyaluminium chloride (PAC)- and biochar reduced NH_3_ emissions by 92, 54, 65 and 77% compared to the slurry control, while lime increased emissions by 114%. Cumulative N_2_O emissions of cattle slurry increased when amended with alum and FeCl_2_ by 202% and 154% compared to the slurry only treatment. Lime, PAC and biochar resulted in a reduction of 44, 29 and 63% in cumulative N_2_O loss compared to the slurry only treatment. Addition of amendments to slurry did not significantly affect soil CO_2_ release during the study while CH_4_ emissions followed a similar trend for all of the amended slurries applied, with an initial increase in losses followed by a rapid decrease for the duration of the study. All of the amendments examined reduced the initial peak in CH_4_ emissions compared to the slurry only treatment. There was no significant effect of slurry amendments on global warming potential (GWP) caused by slurry land application, with the exception of biochar. After considering pollution swapping in conjunction with amendment effectiveness, the amendments recommended for further field study are PAC, alum and lime. This study has also shown that biochar has potential to reduce GHG losses arising from slurry application.

## Introduction

The land application of dairy cattle slurry to farmland can result in incidental and chronic phosphorus (P) losses to a waterbody [1] resulting in eutrophication [2]. Incidental P losses take place when a rainfall event occurs shortly after slurry application and before slurry infiltration into the soil, while chronic P losses are a long-term loss of P from the soil as a result of a build-up in soil test P (STP) caused by application of inorganic fertilisers and manure [1]. Previous studies have indicated that that even with no additional P inputs, it can take up to 20 yr in soils of high STP to reduce to acceptable limits [3,4,5,6]. It is largely accepted that supplementary measures, in particular the development of P mitigating technologies, will be necessary to address the time lag between implementation of these strategies and reduction of STP to the appropriate levels.

Reactive nitrogen (N) losses from slurry also pose significant environmental risks. which is applied in excess of crop requirements can be converted in the soil through mineralization, and processes of nitrification and de-nitrification. Gaseous N loss from slurry due to the volatilisation of ammonia (NH_3_) is the major N loss pathway from slurry, resulting in a 50%-80% loss of total ammoniacal nitrogen (TAN). This represents both a considerable reduction in the N fertiliser value of slurry and a considerable source of atmospheric pollution as ammonia is both an acidifying gas and a source of terrestrial and aquatic eutrophication following deposition [7, 8, 9,10]. In addition, landspreading can increase emissions of greenhouse gases (GHG), particularly nitrous oxide (N_2_O) [11 12], but also carbon dioxide (CO_2_) [13, 14] and methane (CH_4_) [15].

Currently, Irish agriculture accounts for 32.1% of all national GHG emissions [16] and for virtually all NH_3_ emissions [17]. Whilst CH_4_ from enteric fermentation comprises the bulk of greenhouse gas emissions, N_2_O - associated with N inputs to soils—comprise the second-largest source (36.5%). Nitrous oxide, in turn, contributes to both global warming, due to its high global warming potential (296 times that of CO_2_), and also stratospheric ozone depletion [18, 19]. Although NH_3_ is not a GHG, it contributes to acidification of soils, atmospheric pollution and eutrophication of surface and ground water systems [20]. An estimated 5% of global N_2_O emissions results as a consequence of wet and dry deposition of NH_3_ and subsequent nitrification/denitrification [21]. Approximately 40 million tonnes (Mt) of animal manures are produced annually in Ireland resulting in a national emissions of 103.7 kt NH_3_. Landspreading of manures accounts for 35% of this total [16]. However, it is anticipated that these emissions may rise in the future, due to the fact that dairy sector expansion may result from milk quotas being abolished within the European Union. [22].

Chemical amendment of dairy cattle slurry before land application has been proposed as a strategy to mitigate P losses by reducing the solubility of P in slurry [23, 24]. The most effective chemicals at reducing dissolved reactive phosphorus (DRP) from overlying water have been shown to be (from best to worst) alum, poly-aluminium chloride (PAC), ferric chloride (FeCl_2_), and lime, once all associated costs have been taken into account.

Recent research on the use of biochar (pyrolysed organic material) as a soil amendment has shown its beneficial effects on soil fertility [25, 26] and at reducing greenhouse gas emissions [27, 28]. In particular, it may help retain ammonium in fertilizer and lower gaseous N emissions [26, 27]. In addition, biochar has been proposed as a potential P mitigation amendment with a 50% reduction of soluble P and an increase in plant available P reported for dairy slurry lagoons [29]. Therefore, there is potential that certain biochars could be used to mitigate P losses effectively. As there is a large body of work involving biochars being carried out at present and there is the potential in their use for P remediation, the impact of the landspreading of biochar on GHG emissions was also evaluated as part of this study.

When evaluating the feasibility of any amendment to slurry, it is critical that whilst amendments may prevent nutrient loss in the solute phase, the ‘pollution swapping’, defined by Stevens and Quinton [30] as ‘the increase in one pollutant as a result of a measure introduced to reduce a different pollutant’ in the gaseous phase be considered. Therefore, the aims of this study were: (i) to elucidate the effects of chemical treatment on the loss of NH_3_, CH_4_, N_2_O, and CO_2_ from dairy cattle slurry applied to grassland soils and (ii) to further refine the feasibility of using chemical amendment based on their potential for greenhouse warming effects.

## Materials and Methods

### Soil sample collection and analysis

Intact soil samples were taken from a dairy farm (53°21'150 N, 8°34' W) in County Galway. No permission was required at either location as these were research dairy farms located at Teagasc, Athenry and the corresponding author and co-author (Owen Fenton) are both research officers in Teagasc. Aluminium (Al) coring rings, 120-mm-high, 100-mm-diameter, were used to collect undisturbed soil core samples (n = 18). Soil samples, taken to a depth of 100 mm below the ground surface from the same location, were air dried at 40°C for 72 h, crushed to pass a 2 mm sieve, and analysed for Morgan’s P (the national test used for the determination of plant available P in Ireland) using Morgan’s extracting solution [31]. Soil pH (n = 3) was determined using a pH probe and a 2:1 ratio of deionised water-to-soil. Soil texture was determined by particle size distribution [31]. Organic matter (OM) content of the soil was determined using the loss of ignition [32]. The soil was a poorly-drained sandy loam (58% sand, 27% silt, 15% clay) with a Morgan’s P of 22±3.9 mg P L^-1^, a pH of 7.45±0.15 and an OM content of 13±0.1%. Historic applications of organic P from an adjacent commercial sized piggery led to high STP in the soil used in this study.

### Dairy slurry collection and analysis

Cattle slurry from dairy replacement heifers was taken from a dairy farm (53°21’ N, 8°34’ W) in County Galway, Republic of Ireland. No permission was required at either location as these were research dairy farms located at Teagasc, Athenry and the corresponding author and co-author (Owen Fenton) are both research officers in Teagasc. The field studies did not involve endangered or protected species. Before sample collection, the storage tanks were agitated. Samples were transported to the laboratory in 10-L drums and stored at 4°C. The pH of slurry and amended slurry was determined using a pH ProfiLine 3110 probe (WTW, Germany) and the water extractable phosphorus (WEP) of slurry was measured at the time of land application [33]. The total P (TP) of the dairy cattle slurry was determined after Byrne [33]. Potassium (K) and magnesium (Mg) were analyzed using a Varian Spectra 400 Atomic Absorption instrument and analyses for N and P were carried out colorimetrically using an automatic flow-through unit. Ammoniacal nitrogen (NH_4_-N) of slurry and amended slurry was extracted from fresh slurry by shaking 10g of slurry in 200 ml 0.1 M hydrochloric acid (HCl) on a peripheral shaker for 1 h and filtering through a No 2 Whatman filter paper and analysed using an Aquakem 600 Discrete Analyser (Thermo Scientific, Vantaa, Finland).Total C and N content of slurry were analysed using a LECO TruSpec CN analyser (LECO Corporation, St. Joseph, MI, USA).

### Chemical amendment of slurry

Six treatments were examined under laboratory conditions in this study, with treatments selected from Brennan et al. [23, 24]. With the exception of biochar, the amendments were applied at the following stoichiometric rates determined from Brennan et al. [24]: alum 1.11:1 (Al: TP); PAC 0.93:1 (Al:TP); FeCl_2_ 2:1 (Fe:TP); and lime 10:1 (Ca: TP). Biochar, was derived from wood shavings (2 mm diameter) pyrolised in a muffle furnace at 650^°^C for 4.5 hours and was applied at a rate equivalent to 3.96 m^3^ ha^-1^. This rate was selected based on results of a batch experiment to reduce ammonia emissions (Brennan et al., unpublished data). Slurry characteristics for each amendment are detailed in Table 1.

**Table 1:** Dairy cattle slurry and amended dairy cattle slurry properties.

Treatment	DM (%)	pH	WEP (g kg^-1^ DM)
Slurry only	10.5 (0.04)	7.5 (0.05)	1.81 (0.112)
Alum	9.4 (0.16)	5.4 (0.12)	0.008 (0.002)
Lime	8.2 (0.29)	12.2 (0.12)	0.014 (0.001)
FeCl_2_	10.1 (0.22)	6.7 (0.06)	0.017 (0.001)
PAC	9.6 (0.28)	6.4 (0.05)	0.011 (0.002)
Charcoal	12.57 (0.45)	7.3 (0.4)	1.78 (0.23)

The amendments were added to the slurry and mixed rapidly using a blender immediately before simulated land application. Slurry and amended slurry were applied directly to the surface of the intact grassed soil at a rate equivalent to 33 m^3^ slurry ha^-1^. Immediately after application, the chambers were sealed and the air flow through the system was started and maintained for 168 h.

### Measurement of ammonia (NH_3_)

**Fig 1:**
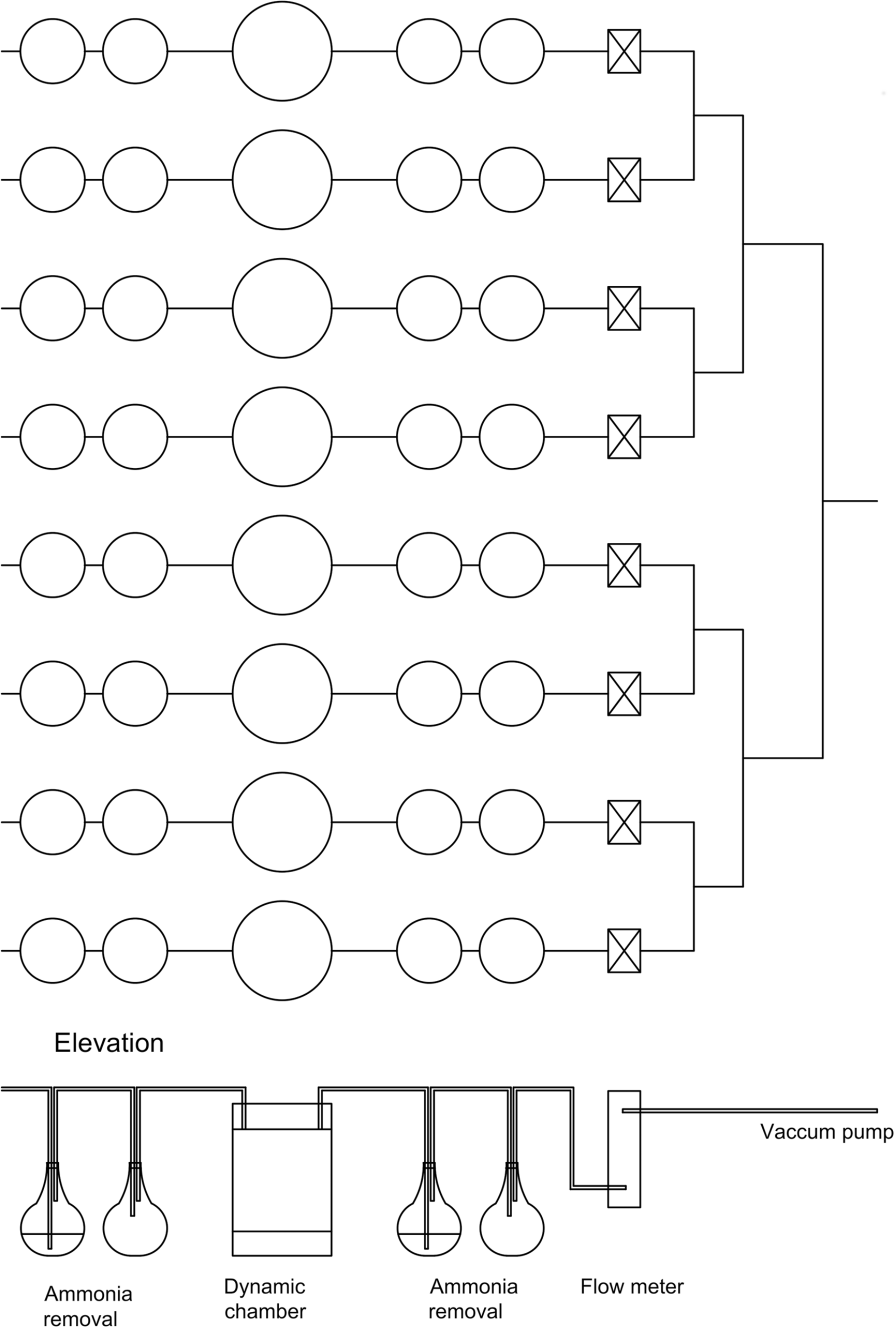
Diagram of apparatus.

**Fig 2:**
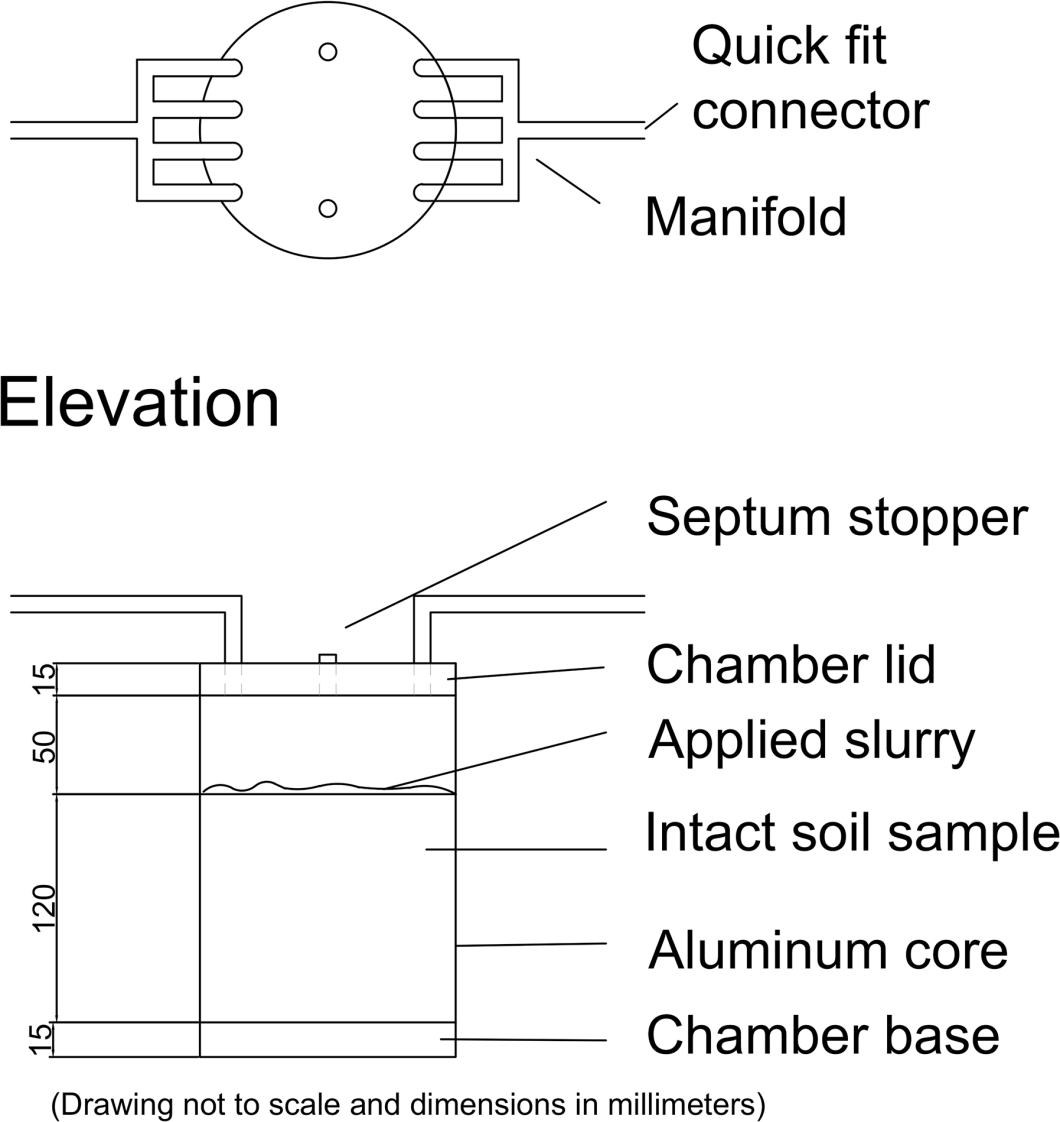
Schematic of the dynamic chambers.

Soil chambers comprised the same 200-mm-diameter aluminium casings used to collect the grassed soil samples, fitted with a polypropylene (PP) lid and base (Figs 1 and 2). The samples were saturated for 48 h and then allowed to drain for 48 h under laboratory conditions. During this time, the surfaces were covered to avoid evaporation losses. After approximate field capacity was achieved, the chambers were sealed at the base using silicon grease to ensure an air-tight seal. Each treatment was applied to the grassed-soil surface and a lid was fitted to each chamber. Each chamber had four inlet and outlet ports to ensure good mixing of air within the chamber.

The dynamic chamber used in this experiment consisted of an open dynamic chamber system, with ammonia concentrations measured at the inlet and outlets to the chambers. Eight chambers per treatment were connected in parallel (Fig 1). Air was drawn through the system via a vacuum pump, (VTE 10 vacuum pump, Irish Pneumatic Service Ltd., Ireland) with air flow through each chamber regulated at 5.1 L min^-1^ using gas mass flow meters at the inlet and outlet (Cole-Parmer, Hanwell, UK).

The air flow regulation ensured that the emission of NH_3_ would not be affected by small differences in flow rates between chambers [32]. Ammonia contained in the air at both the inlets and outlets of the chambers was immobilised by acid trapping method. This involved bubbling the air through conical flasks containing 3% oxalic acid in an acetone solution.

The cores were attached to the dynamic chamber for 168 hours, with flasks replaced after 1, 2, 6, 24, 48, 96 and 168 hours The majority of NH_3_ volatilisation arising from spreading of slurry occurred in initial 48 h after spreading. Therefore, it was only necessary to use the acid-trap system for the first 168 h. Fluxes were subsequently calculated as the differential between the inlet and outlet NH_3_ concentration, accounting for the mass flow of air across the chamber per unit time [34].

### Measurement of CH_4_, N_2_O and CO_2_

Methane (CH_4_), nitrous oxide (N_2_O) and carbon dioxide (CO_2_) were measured by re-configuring the chambers to a dynamic closed system. Air samples were drawn from the head space of the chamber and circulated into a photo-acoustic-analyser (PAA; INNOVA 1412, Lumasense Inc, Denmark) which analysed for CH_4_, CO_2_ and N_2_O. Water vapour was scrubbed prior to entering the analyser by placing a tube containing a mixture of magnesium perchlorate and glass beads in line at the outlet. This prevented water vapour interference within the portion of the infra-red spectrum where methane optimally absorbs (1254 cm^-1^). Subsequently the air was vented back into the chambers. Therefore, the flux could be calculated as the increase in gas concentration as a function of time. During the first 168 h (during which time NH_3_ was measured), each chamber was disconnected from the ammonia acid-trap apparatus, and the inlet and outlet tubes connected to the PAA in a closed circuit. Gas was circulated between the chamber and analyser for 10 min at t = -1 (1 hr before treatment), 0, 2, 6, 24, 48, 72, 96, 144, 168. Fluxes were calculated from the concentration increase of each gas over this period. After 168 h, NH_3_ measurement was discontinued and the chambers, containing intact soil samples, were removed from the apparatus and incubated in the laboratory. During this time, a portable cap was fitted to each chamber and the PAA was used to measure fluxes over a 15 minute period at t = 9, 11, 13, 15 and 17 d. The mass of the sample, and therefore water content, was kept constant throughout the experiment by periodically adding deionised water to the surface of the soil samples.

### Pollution swapping and feasibility

All greenhouse gas emissions were converted to CO_2_ equivalents (IPCC, 2006). The ranking system, determined by Brennan et al. [23], was based on effectiveness of amendments, efficiency of amendments, cost of sourcing and addition of amendments to slurry and potential barriers to use. This study incorporated the greenhouse warming potential (GWP) of each of the amendments into this ranking system. The amendments were ranked from best to worst in decreasing order to enable a combined feasibility score to be calculated. In terms of ranking the pollution balances a weighting system can be applied, whereby the weighting of trade-offs between different loss pathways can be judged (Eq 1). The advantage of this is that different regions can apply different weightings depending on the environmental policy emphasis. An example here is where aeration is a problem in a system, resulting in a trade-off between CH_4_ and N_2_O or N_2_O and NH_3_. Ammonia was classified separately rather than expressing it in terms of an indirect greenhouse gas, due to the fact that it has multiple impacts.

$$x=a\left(B_{N2O}\right)+B\left(B_{NO3}\right)+c\left(B_{CH4}\right)+d\left(B_{CO2}\right)+etc$$ (1)

where a-d and so on are weighting factors and B is the cumulative loss of a given pollutant over a pre-defined period (eg. one year). Other contaminants in gaseous (e.g. NH_3_ and H_2_S), dissolved (e.g. ammonium, metals) and particulate (e.g. particulate P) forms may also be added.

### Statistical analysis

Data were checked for normality and homogeneneity of variance by histograms, qq plots, and formal statistical tests as part of PROC UNIVARIATE procedure of SAS [35]. The data were analysed using the PROC GLM procedure [35]. This analysis used a linear model which included the fixed effects of treatment. With the exception of slurry pH, NH_3_, CH_4_ and CO_2_, data were logarithmically transformed prior to analysis. A multiple comparisons procedure (Tukey) was used to compare means.

## Results

### Slurry and amended slurry results

The slurry had total N (TN) of 4430±271 mg L^-1^, total P of 1140±76 mg L^-1^, total K (TK) of 4480±218 mg L^-1^, and a pH of 7.5±0.05. The slurry TP and TK remained relatively constant, while the WEP was lowered significantly by all chemical amendments (Table 2). Alum, FeCl_2_ and PAC addition reduced slurry pH from approximately 7.5 to 5.4, 6.7, and 6.4, respectively (*p*<0.005). The pH of alum-amended slurry was significantly different to all other treatments, while FeCl_2_ and PAC were not significantly different to each other. Addition of lime increased slurry pH to 12.2 (*p*<0.001), while charcoal did not have a significant effect on slurry pH.

**Table 2:** Cumulative CH_4_, CO_2_, N_2_O and NH_3_ emissions from amended and control slurries for 15 days post-application.

Treatment	CH_4_ (kg CH_4_-C ha^-1^)		CO_2_ (kg CO_2_-C ha^-1^)		N_2_O (kg N_2_O-N ha^-1^)		NH_3_ (kg NH_3_-N ha^-1^)	
	mean	std	mean	std	mean	std	mean	std
Slurry only	8.45a	8.29	440.4a	11.2	0.39a	0.13	28.5a	2.52
Alum	7.62a	2	526.5a	6.2	1.18b	0.78	2.46b	2.01
FeCl2	-0.75b	0.98	467.4a	71	0.99b	0.17	13.2a	1.02
Lime	-2.88b	0.77	489.4a	69.1	0.2a	0.1	60.3a	10.3
PAC	-2.0 b	0.29	457.3a	66.6	0.3a	0.08	1.07b	3.19
Biochar	0.1 b	0.2	72.7b	44.1	0.1a	0.02	7.36b	3.38

Letters indicate least significant difference (P<0.05) between treatments.

### Ammonia

**Fig 3:**
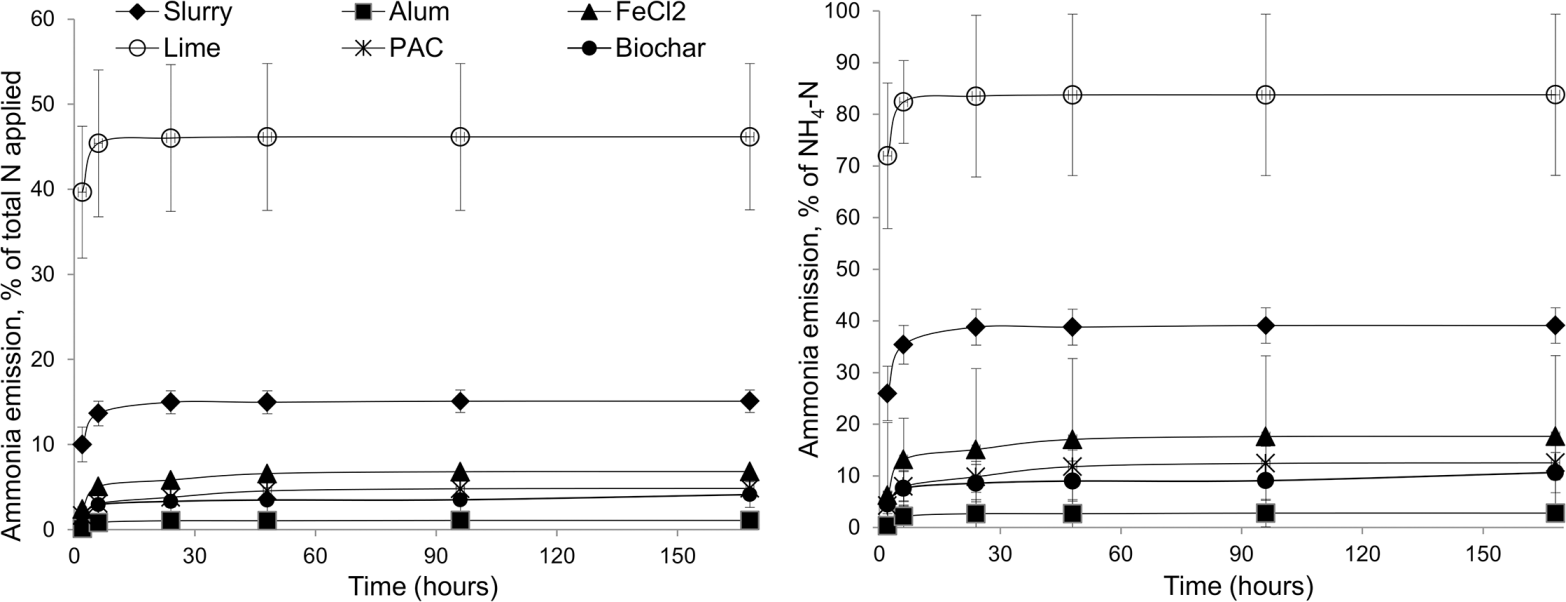
Ammonia emissions. Emissions from untreated and chemically amended slurry expressed as a percentage of total nitrogen in slurry and total ammoniacal nitrogen in slurry.

Alum), FeCl_2_ PAC and biochar significantly reduced NH_3_ emissions by 92, 54, 65 and 77% compared to the slurry control (p<0.01), while lime increased emissions by 114% (*p*<0.01, Table 2). Lime amendment resulted in the loss of 84% of TAN applied. Alum, PAC, FeCl_2_ and char were not statistically different to each other. The NH_3_ emissions from broadcast-applied untreated and chemically amended slurry, expressed as a percentage of TN and NH_4_-N in the applied slurry, are shown in Fig 3. Ammonia release from slurry for all treatments followed a Michaelis-Menten response curve, with the majority of emissions occurring within the first six hours following application. With the exception of the lime treatment, chemical amendment of slurry prior to land application increased the time for half of ammonia losses to occur (T_0.5_). Alum, FeCl_2_, PAC and biochar all increased T_0.5_ significantly (p<0.001) compared to the slurry control, from 1.5 to 4.1, 3.5, 4.3 and 3.4 h, respectively. The T_0.5_ of lime-amended slurry was not significantly different to the slurry control. Cumulative ammonia release from untreated slurry was 40% of TAN.

### Nitrous oxide

**Fig 4:**
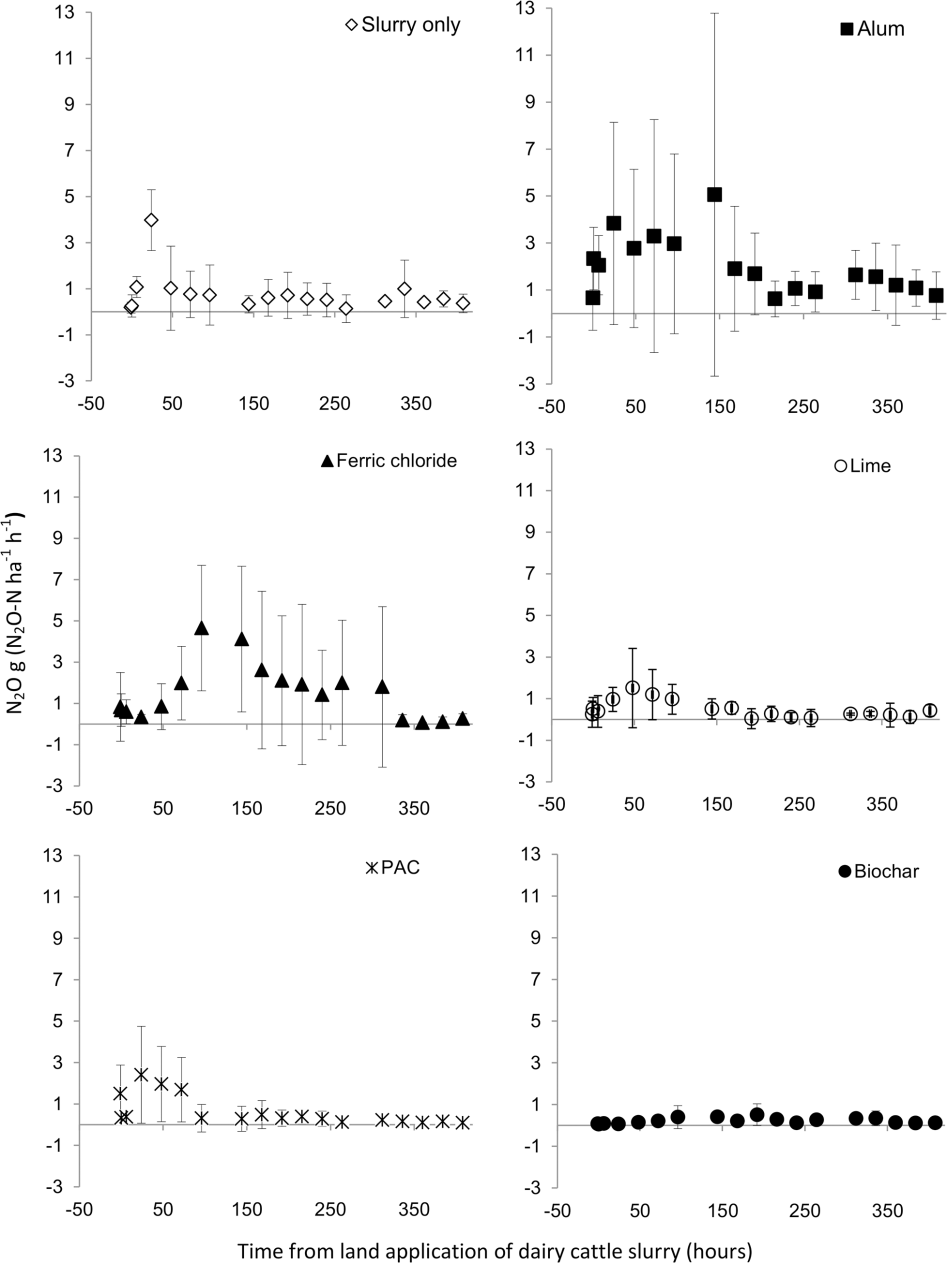
Nitrous oxide emissions profile. Temporal profile of nitrous oxide (N_2_O-N) emissions from amended slurry and control treatments for 15 days after application.

Cumulative N_2_O emissions of dairy cattle slurry increased when amended with alum and FeCl_2_ by 202 and 154% respectively compared to the slurry control. In contrast, lime and biochar resulted in a reduction of 44%, and 63% in cumulative N_2_O loss compared to the slurry control while PAC did not have a significant effect on N_2_O emissions (Table 2). In this study, nitrous oxide emissions following land application of dairy cattle slurry were observed to increase from background levels of 0.18 g N_2_O-N ha^-1^ h^-1^ to a peak of 4 g N_2_O-N ha^-1^ h^-1^ at 24 h post-application (Fig 4). Emissions of N_2_O from alum were similar in magnitude and temporal dynamics to those from the slurry control. Ferric chloride addition resulted in no increase in N_2_O emissions until the 72 h sampling event, and a peak flux of 4.7 g N_2_O-N ha^-1^ h^-1^ was measured at 96 h. Lime, PAC and biochar addition resulted in much lower emissions, with peak emissions occurring after 24–48 h.

### Carbon dioxide

**Fig 5:**
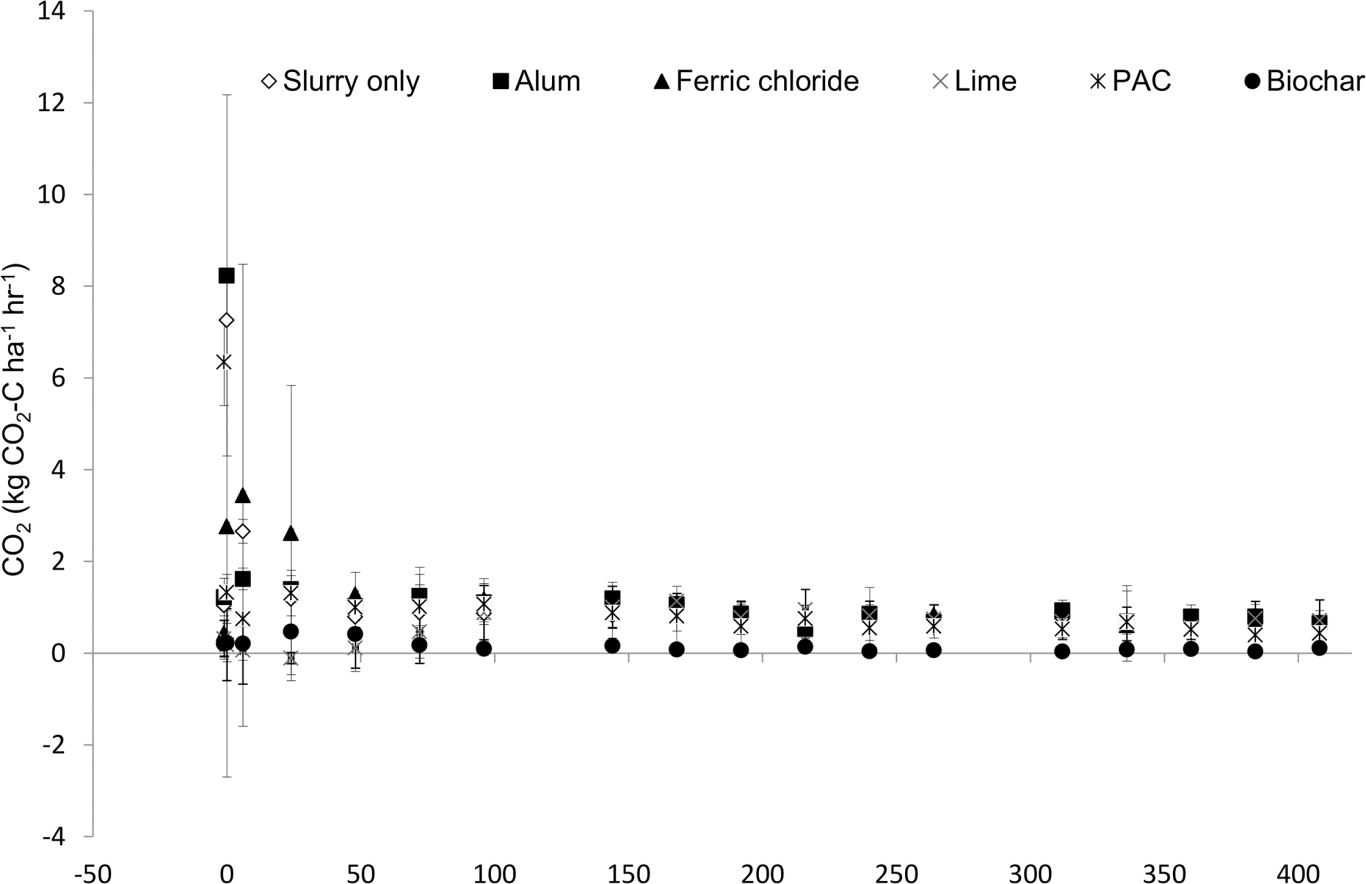
Carbon dioxide emissions profile. Temporal profile of carbon dioxide (CO_2_-C) emissions from amended slurry and control treatments for 15 days after application.

In general, addition of amendments to slurry did not significantly affect soil CO_2_ release during the study (Fig 5), with cumulative emissions for the period ranging from 420–480 kg CO_2_ ha^-1^ (Table 2). However, significant reductions in CO_2_ efflux were observed upon biochar addition, with an 84% reduction in cumulative CO_2_ emissions observed (*p*<0.05). Immediately following land application of dairy cattle slurry and chemically amended slurry, there was generally a peak in CO_2_ emissions followed by a steady release for the duration of the study. The lime amended slurry behaved differently to the other treatments and the slurry control, and acted as a CO_2_ sink immediately after land application. However, the cumulative emissions were similar to PAC and FeCl_2_ treated slurry.

### Methane

**Fig 6:**
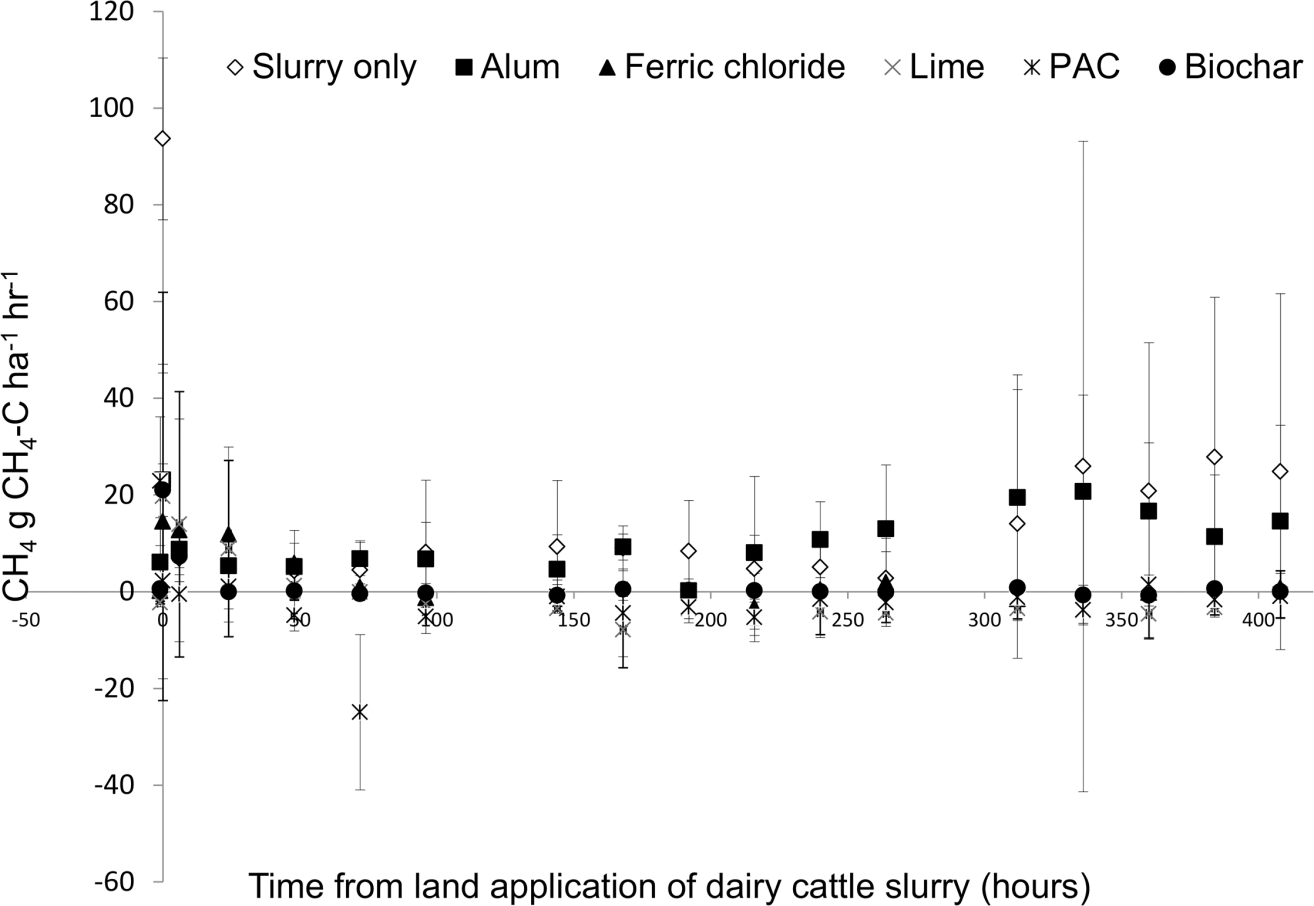
Methane emissions profile. Temporal profile of methane (CH_4_-C) emissions from amended slurry and control treatments for 15 days after application.

Methane emissions increased from -0.18 g CH_4_-C ha^-1^ h^-1^ to 94 g CH_4_-C ha^-1^ h^-1^ upon application of dairy cattle slurry (Fig 6). These levels decreased rapidly to approximately 7 g CH_4_-C ha^-1^ h^-1^ by 48 h and remained relatively constant until the 312 h sampling event. Following this, methane losses were much more variable. There was a similar trend for all of the amended slurries applied with an initial increase in losses followed by a rapid decrease and then steady release for the duration of the study. All of the amendments examined reduced the initial peak in CH_4_ emissions compared to the slurry control (*p*<0.05). Lime significantly reduced cumulative CH_4_ emissions by 134% (*p*<0.05, Table 2). PAC and FeCl_2_ (*p*<0.09) also reduced cumulative CH_4_ emissions compared to the slurry control by 121 and 99%, respectively. However, these reduction were not significant (p = 0.08 and p = 0.09 respectively). Alum, and biochar had no significant effect on emissions compared to the slurry control.

### Impact of amendments on global warming potential

**Fig 7:**
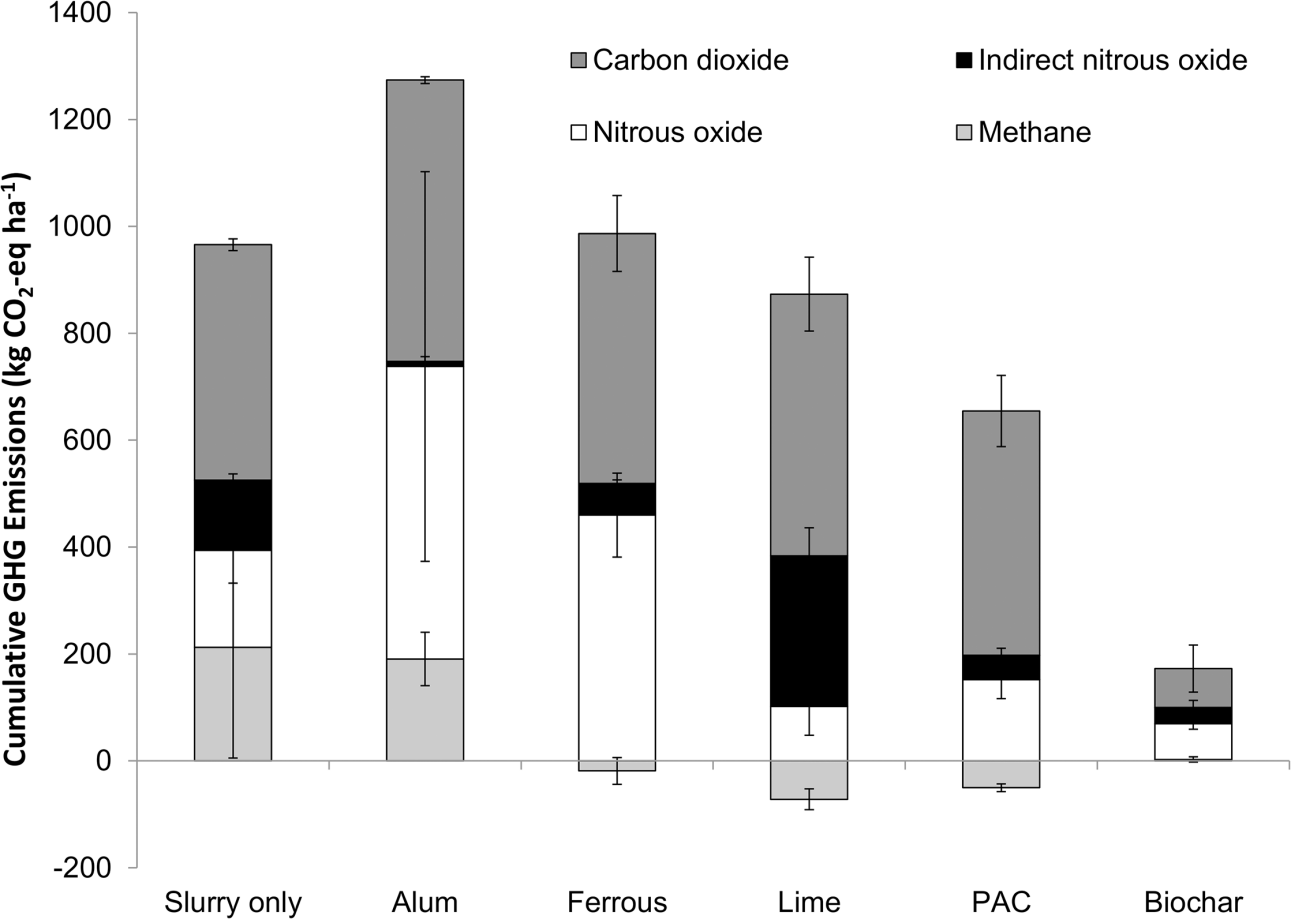
Cumulative greenhouse gas emissions. Total emissions (expressed as CO_2_- equivalents) for 15 days post-application of amended and control slurries.

Chemical amendment of dairy cattle slurry has been proposed as a possible P mitigation measure for the control of P solubility in dairy cattle slurry [23, 24]. In order to access the pollution swapping potential of the treatments, all emissions were expressed in CO_2_ equivalents. Cumulative direct and indirect N_2_O emissions from slurry and amended slurry in the chambers during the study are shown in Fig 7. Indirect N_2_O emissions were calculated based on the assumption that all the NH_3_ would be re-deposited within a 2 km radius of the point of application, which allowed use of an emission factor of 1% [19]. Alum, FeCl_2_, lime and PAC have no significant effect on the sum of the cumulative direct and indirect N_2_O emissions, while charcoal reduced total N_2_O emissions by 69% compared to the slurry control (*p*<0.01). The total N_2_O emissions from charcoal treated slurry–with the exception of PAC—were statistically different to slurry (*p*<0.01), alum (*p*<0.01), FeCl_2_ (*p*<0.01), lime (*p*<0.01) treatments. Cumulative carbon dioxide and methane emissions are shown in Fig 7. Biochar reduced total cumulative CO_2_ and CH_4_ emissions compared to the control (*p*<0.01) and was significantly different (p<0.05) compared to alum, FeCl_2_), lime and PAC. Amendment of slurry with biochar significantly reduced GWP following land application of dairy cattle slurry (*p*<0.01). In this study, there was no significant effect of any amendment of slurry on GWP caused by land application dairy cattle slurry, with the exception of biochar.

## Discussion

In general, P amendments affected gaseous emission by either altering slurry pH or increasing immobilisation. For instance, lime, PAC and biochar addition resulted in a reduction in N_2_O emissions, but while the reductions associated with PAC and biochar were most likely due to N immobilisation, the lack of N_2_O associated with lime application was due to increased ammonia loss. This demonstrated the need to quantify all loss pathways when evaluating amendments.

### Ammonia emissions

Ammonia volatilisation from dairy cattle slurry following land application is influenced by humidity, temperature, wind speed, method of application, and the degree of infiltration of the slurry into the soil [7,8, 9, 36,37, 38]. In addition, slurry pH, DM and TAN content greatly influence the rate and amount of NH_3_ volatilisation [7, 8, 9 39]. It is estimated that between 60–80% of TAN applied can be lost during broadcast land spreading of cattle slurry, particularly during the first 12 h post application [22, 40]. In the present study, cumulative NH_3_ loss from land applied dairy cattle slurry was 22.6 kg NH_3_-N ha^-1^, with approximately 39% of NH_4_-N applied lost in initial 24 h; this was equivalent to 15% of total nitrogen (TN) applied.

With the exception of lime, all amendments used reduced NH_3_ losses compared to the slurry control. This reduction was expected as chemical amendments, such as alum, have been used extensively in the USA to reduce NH_3_ emissions from poultry litter [41] and from dairy cattle slurry (Table 3). A 60% reduction in NH_3_ loss from dairy cattle slurry was reported when 2.5% by weight of alum was added in a laboratory batch experiment [39], while in a field study, a 92% reduction in NH_3_ loss was observed in a field study where alum had been applied [42]. The results of the present study were in agreement with previous findings for alum, PAC and FeCl_2_, and the ammonia abatement by alum, PAC and FeCl_2_ was primarily due to reductions in pH (ie. N was held in the ammonium form).

**Table 3:** Summary of amendments used to reduce ammonia emissions in previous studies.

Reference	Chemical	Amount added	Slurry type	Study	%reduction	pH	Comments
Meisinger et al. (2001)	alum	2.5% (w/w)	Dairy	Lab	60	4.5	Simulated storage experiment
	zeolite	6.25% (w)			55	7.8	
Kai et al. (2007)	H_2_SO_4_	5 kg m-3	Swine	Field	70	6.3	Farm scale storage and application
Smith et al. (2001)	Alum	0.75% (v/v)	Swine	Plot	52		6-week study
Molloy and Tunney (1983)	FeSO_4_	0.8 g to 25 g	Dairy	Batch	81		Batch scale experiment
	MgCl_2_	0.8 g to 25 g			23		
	CaCl_2_	0.8 g to 25 g			50	7.8	
Shi et al. (2001)	Alum	4500 kg/ha	Dairy	Field	92	5.98*	Applied to surface of feedlot
	CaCl_2_	4500 kg/ha			71	6.99*	
Husted et al. (1991)	HCL	240 mEq	Dairy		90		
	CaCl_2_	300 mEq		Lab	15		

*pH mentioned here is pH of soil and slurry mixture.

The large reductions in ammonia emissions associated with biochar addition (74%) may have been due to both ammonia gas and ammonium ion adsorption, as biochar can act as a cation exchange medium [43]. During pyrolysis of woody material for biochar production, thermolysis of lignin and cellulose occurs, exposing acidic functional groups, such as carboxyl groups. This has been shown to result in an 80% 100% removal efficiency for ammonia gas [44, 45]. Biochar addition during the composting of poultry litter reduced ammonia losses by 64%, even though pH increased [46]. As a result, the mechanism was thought to be due to the adsorption of ammonium ions as opposed to the immobilization of ammonia [47]. In addition, biochar has also been found to reduce N leaching by 15% due to adsorption of the ammonium ion predominantly by cation exchange [48].

**Fig 8:**
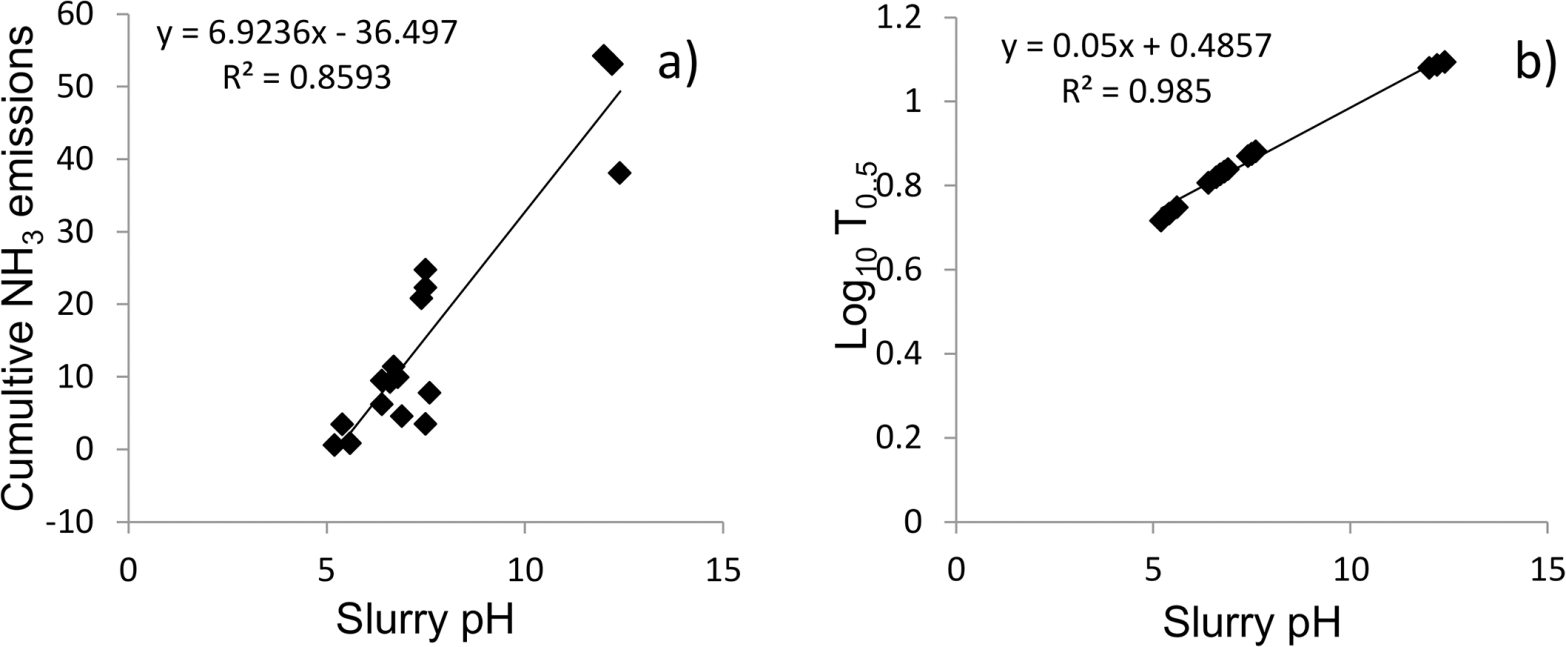
Correlation between ammonia and time. Relationship between slurry and amended slurry pH at time of application and (a) cumulative NH_3_ emissions and (b) and log of time for half of ammonia emissions to occur (T_0.5_).

Lime increased slurry pH to 12.2 and increased the NH_3_ loss compared to the slurry control. Indeed, modeled results have estimated that NH_3_ emissions increase by 3.3 kg ha^-1^ for each increment of 0.1 pH [49]. There was a linear relationship between slurry pH at time of application and NH_3_ loss from slurry and amended slurry in this study (R^2^ = 0.86) (Fig 8). This would indicate that the change in slurry pH was the main process responsible for the reduction in NH_3_ loss from dairy cattle slurry. In addition, there was a significant relationship (R^2^ = 0.98) between slurry pH at time of application and the log of the T_0.5_ (Fig 8). This would indicate that if large NH_3_ losses do not occur in the short term after land spreading, the potential for loss is significantly reduced i.e. chemical treatments are not just delaying NH_3_ loss, but mitigating it completely.

In addition to environmental problems caused by NH_3_ losses, such losses reduce the nutrient value of the fertiliser and increase NH_3_ emissions from slurry. The value of N lost via ammonia and N_2_O emissions from the slurry control for the duration of the study amounted to approximately €0.63 per m^3^ slurry applied based on cost of €1.10 per kg N [50]. Alum, FeCl_2_, PAC and biochar increased the fertiliser value of slurry by €0.56, €0.32, €0.41 and €0.48 per m^3^ slurry compared to the slurry control.

### Nitrous oxide

Land application of agricultural wastes results in an increase in N_2_O emissions from soil) and these emissions are influenced by: soil moisture status, soil temperature; soil nitrate (NO_3_) content and organic carbon content [51, 52]. It was hypothesised that any reduction in NH_3_ loss would result in a concomitant increase in soil–derived N_2_O due to higher mineral N available for nitrification/denitrification. Whilst there were no significant differences due to large standard deviations within treatments, this general trend was observed for both alum and ferrous chloride treatments where large reductions in ammonia emissions were offset by a doubling in cumulative N_2_O losses compared to the slurry only treatment. Also, whilst lime addition resulted in a decrease in *direct* N_2_O losses, these were merely due to the fact that most of the available mineral N had been already lost during volatilisation. Indeed, only biochar addition significantly lowered emissions (*p*<0.01) relative to the slurry control. Ammonia volatilisation can also lead to indirect N_2_O emissions as the majority of ammonia volatilised in the field is re-deposited within 2–5 km *via* wet and dry deposition, and a proportion (1%) is re-emitted as N_2_O [19]. When these indirect losses were calculated, lime addition accounted for an increase in indirect N_2_O emissions from 283 g N_2_O ha^-1^ for the slurry control to 606 g N_2_O ha^-1^. These results highlight the need to account for all gaseous N losses as an analysis of ammonia or N_2_O in isolation would give skewed results.

In terms of abating total N emissions, char was the most effective with losses reduced by 63%. Other studies comparing biochar and hydrochar effects on soil GHG emissions have observed a 40–50% reduction in N_2_O release [27, 28]. However, this abatement potential is highly dependent on the source material of the char and the temperature of pyrolization [28, 53]. The rate of char application may be less important, with studies comparing 1% and 3% biochar incorporation reporting similar levels (50%) of emission reduction [28]. The mechanism for N_2_O reduction is unclear. Some studies have indicated that biochar may reduce N_2_O by increasing soil aeration and hence reduce water-filled pore space [54]. Also adsorption of ammonium (NH_4_^+^) or nitrate (NO_3_^-^) onto the charcoal surface has been hypothesised [44]. Alternatively, if pH is increased upon char addition, this may induce a shift towards total de-nitrification to N_2_, thus reducing N_2_O [55].

### Carbon emissions

There was no significant change soil CO_2_ respiration upon amendment addition, with the exception of biochar, where a significant reduction in CO_2_ emissions was observed. Previous reported effects of char application on CO_2_ efflux are varied. Biochar application to organic manures has shown an increase in C emissions in the short term [46], while biochar addition to soils have also indicated a simulation of soil microbial respiration [56]. A comparison on the effect of 16 biochars on CO_2_ emissions reported increases and decreases in emissions, depending both feedstock, method and temperature of pyrolysis [53]. Similarly, a 90% reduction in soil respiration was observed upon the addition of wood-derived char to soil whilst the addition of hydrochars stimulated CO_2_ release [27]. The differences between previous reports are possibly due to variations in the proportion of labile C available on the char and the mineralization of carbonate groups on the surface of the biochar. Suppression of soil respiration upon char addition is most likely, therefore associated with either sorption of CO_2_ onto the biochar or a reduction in labile C availability.

After land application, CH_4_ emissions are of minor importance compared to NH_3_ and N_2_O emissions [57]. Methane is produced mainly by microbial decomposition of organic matter under anaerobic conditions. The highest efflux was for untreated slurry and alum, immediately post manure application would indicate CH_4_ formation during manure storage, as there would not be sufficient time for its formation in the soil. It is produced during slurry storage and shortly after slurry application, after which time the organic matter is oxidised to CO_2_ and H_2_O as aerobic conditions prevail. Initial CH_4_ emissions in the following few hours most likely originate from CH_4_ contained in the manure diffusing from the viscous layer, while subsequent emissions were likely to be produced during the degradation of labile C compounds [58 59]. Similar base-line CH_4_ soil emission levels of 1.1 kg CH_4_-C ha^-1^ day^-1^ (3.01 g CH_4_-C ha^-1^ day^-1^) have been observed from Swedish cereal cropped soils [60, 61] reported, while similar peaks CH_4_ emissions of approximately 75 g CH_4_-C ha^-1^ day^-1^ immediately post application of cattle slurry to grassland have been recorded [58]. High emission levels following pig and dairy manure application to grassland soil in laboratory experiments have also been reported [15].

Biochar suppressed CH_4_ emissions upon slurry landspreading. Amendment of biochar to wastewater sludge has previously been shown to have no effect on methane release [28] and indeed biochar addition to soils have been shown to reduce oxidation of methane [27]. However, this may be soil-specific with highly organic soils that are prone to methane emissions, exhibiting a decrease in emissions [51]. In general, the effect of biochar on methane release and/or uptake appears to be variable.

### Impacts of pollution swapping

Whilst the efficacy of the various slurry amendments on P sequestration efficiency is well quantified [23, 24, 62, 63], there is less information on their effects on other loss pathways, particularly gaseous emissions. This study gives a much needed consideration to the risk of amendments on gaseous losses which are critical in selecting amendments for recommendation to legislators. The study also allowed for the effect of chemical amendment on gaseous emissions to be incorporated into the feasibility analysis of Brennan et al. [23]. A new feasibility analysis was developed to include the results of this study and to give recommendations for the best amendment to mitigate DRP losses with the least potential for pollution swapping. The results of this feasibility analysis are shown in Table 4. Biochar was excluded as there is insufficient data on P sequestration potential to date. In order of decreasing feasibility, the amendments were ranked from best to worst as follows: PAC, alum, FeCl_2_ and lime. Therefore, the amendments selected for recommendation for further study are from best to worst: PAC, alum and lime. Ferric chloride was excluded due to risk of stability of Fe-P bonds in soil. Although there are similar concerns with lime, it is currently added to soil in Ireland to reduce acidity in soils and for this reason, it was decided to recommend lime over FeCl_2_.

**Table 4:** Summary of feasibility of amendments (Adapted from Brennan et al. (2011)).

Chemical	Ratio used Brennan et al. (2011a)		Feasibility score P	Pollution swapping score	Feasibility score	Notes
Alum	0.98:1 Al: P		1	5	6	Risk of effervescence
						Risk of release of H_2_S due to anaerobic conditions and reduced pH
						Cheap and used widely in water treatment
						Reduced ammonia emissions
PAC	0.98:1 Al: P		2	2	4	No risk of effervescence (Smith et al., 2004)
						AlCl_3_ increased handling difficulty
						Expensive
						Reduced ammonia emissions
FeCl_2_	2:1 Fe: P	3		4	7	Potential for Fe bonds to break down in anaerobic conditions
						Increased release of N_2_O
						Reduced ammonia emissions
Ca(OH)_2_	5:1 Ca: P	4		3	7	Increased NH_3_ loss
						Strong odour
						Hazardous substance
Biochar	1					Potential to reduce P solubility limited work to date
						Improve soil microbial health
						Reduced GHG emissions
						Reduced ammonia emissions

Marks for feasibility and pollution swapping are from 1 to 5. 1 = best 5 = worst.

## Conclusion

This study has shown that some P mitigating amendments (Alum, FeCl_2_ and Lime) may result in pollution swapping in terms of gaseous emissions, whilst PAC may be effective in terms of abating both P and gaseous N losses. It also highlights the need to assess the trade-offs on a suite of emissions, particularly NH_3_ and N_2_O. In addition, there is a need to examine the effect of amendments using different soil types under different climatic conditions. This study has also shown that biochar has excellent potential to reduce total gaseous losses arising from land application of dairy cattle slurry. There is a need to develop biochars which are efficient in sorbing P and can improve soil quality and reduce GHG emissions. In addition, at the current cost of treatment, the increase in fertiliser value of the slurry due to some treatments is not sufficient to offset the cost of treatment.
